# Analysis of miRNA-mRNA Crosstalk in Radiation-Induced Mouse Thymic Lymphomas to Identify miR-486 as a Critical Regulator by Targeting IGF2BP3 mRNA

**DOI:** 10.3389/fonc.2020.574001

**Published:** 2021-02-22

**Authors:** Hainan Zhao, Suhe Dong, Jicong Du, Penglin Xia, Ruling Liu, Tingting Liu, Yajie Yang, Ying Cheng, Jianming Cai, Cong Liu, Fu Gao, Hu Liu

**Affiliations:** ^1^Department of Radiation Medicine, Faculty of Naval Medicine, Second Military Medical University, Shanghai, China; ^2^Department of Radiology Intervention, Changhai Hospital Affiliated to the Second Military Medical University, Shanghai, China; ^3^PLA Rocket Force Characteristic Medical Center, Beijing, China; ^4^College of Basic Medicine, Second Military Medical University, Shanghai, China

**Keywords:** ionizing radiation, radiation-induced thymic lymphoma, miR-486, miRNA-mRNA regulatory network, IGF2BP3

## Abstract

Ionizing radiation is one of the common environmental carcinogens. miRNAs play critical roles in the processes of tumor occurrence, development, metastasis. However, the relationship between radiation-induced carcinogenesis and miRNA rarely reported. This study is aimed to investigate the effect of miRNAs on radiation-induced carcinogenesis. In this study we established the radiation-induced thymic lymphoma mice model. By using miRNA array of RTL tissue and predicting for miRNAs target genes, a miRNA-mRNA crosstalk network was established. Based on this network, we identified a critical miRNA, miR-486, which was the most down-regulated in the radiation-induced carcinogenesis. Then the function of miR-486 was confirmed by using knockout mice and cellular experiments. As a result, miR-486 could inhibit proliferation of mouse lymphoma cells by targeting IGF2BP3 mRNA. The adenovirus over-expression miR-486 vector reduced tumorigenesis *in vivo*. MiR-486 knockout mice have a strong tendency of radiation-induced carcinogenesis. In conclusion, miR-486 inhibits the proliferation of lymphoma cells and tumorigenesis induced by radiation through targeting IGF2BP3.

## Background

Carcinogenesis could be induced by environmental factors which mainly include physical, chemical, and biological factors, and ionizing radiation is one of the common physical carcinogens (1–3). Radiation-induced carcinogenesis is the late effects of irradiation exposure and shares a very complicated process involving genomic instability and abnormal signal transduction pathways (4–6). Radiation-induced carcinogenesis has been studied in our lab since 1999, and we successfully established a radiation-induced thymic lymphoma (RTL) mice model by using fractionated irradiation. Using this model, a series of studies have been conducted on the mechanism of radiation-induced carcinogenesis (7).

In recent years, the miRNA is followed with powerful interests. miRNAs play critical roles in the processes of tumor occurrence, development, metastasis, and miRNA generation (8, 9). However, the relationship between radiation-induced carcinogenesis and miRNA expression is rarely revealed. Benefited by the model of RTL, we had reported the roles of MiR-21 (10) and MiR-467 (11) an RTL since 2011. However, the critical miRNAs are still unclear.

With the development in molecular biology and bioinformatics, more and more studies have verified the roles of miRNAs in cancer. The identification of miRNA-mRNA regulatory modules has proven to be important for understanding cellular systems. It is already found that one miRNA could target multiple mRNAs meanwhile one mRNA could be regulated by several miRNAs (12). Therefore the identification of critical miRNAs in RTL may depend on a mass of miRNA/mRNA data and exact bioinformatics analysis.

In this study, we performed genome-wide mRNA and miRNA expression profiling studies between RTL and normal thymus tissues. By constructing regulatory network between miRNAs and their targets, we screened out the miR-486 as the potential regulator in the process of RTL. Further, the effect of miR-486 on mouse lymphoma cells was investigated and its target gene was identified.

## Materials and Methods

### Mice and Treatment

Four-week-old female BALB/c mice (Chinese Academy of Sciences, Shanghai), China) were housed in a specific pathogen free facility. The RTL model and ^60^Co γ-ray whole body irradiation were described as our previous work (10). In short, un-anaesthetized mice were placed in well ventilated plastic boxes and exposed to ionizing radiation. The RTL model was induced by ^60^Co γ-ray whole body irradiation with fractional dose of 1.75 Gy. The total irradiation dose was 7.0 Gy dividing into four times and dose rate approached to 0.58 Gy/min. The split irradiated mice (n = 400) and non-irradiation group (n = 100) were examined daily. Subsequently, RTL tissues and parallel non-irradiated thymus tissues were randomly selected as paired samples. RNAs and proteins were extracted to quantify their expression.

### Sample Processing

Once establishment of RTL, the mice were sacrificed after being anaesthetized with 1% pentobarbital sodium. Then the tissues of RTL and normal thymus were removed to divide into three portions. One portion was placed in 10% formalin, and subsequently was performed H&E staining and immunohistochemical staining. Another part of samples was extracted RNA and proteins to detect their expression by RT-PCR and Western blotting. The remains were conducted Affymetrix miRNA chip and mRNA sequencing.

### Cell Culture and Transfection

Mouse lymphoma cells line (EL4), obtained from Chinese Academy of Sciences (Shanghai, China), was cultured at 37°C in a humidified atmosphere of 5% CO_2_ with 1640 Medium (PAA Laboratories) containing 10% fetal bovine serum (PAA Laboratories). MiR-486 mimics, miR-NC mimics (miR-negative control mimics), miR-486 ASO (miR-486 antisense oligo), and miR-NC ASO (miR-negative control antisense oligo) were obtained from Gene pharma company (Shanghai, China). The cells were planked originally in the 24 wells plate with density of 0.5–2 × 10^5^/well. When the planked cells reach to 80%, miRNAs were transfected using Lipofectamine 2000 (Invitrogen). Specific procedure of transfection was in accordance with previous study (13).

### Cell Viability Test

The cell viability was detected by CCK-8 assay. Firstly, EL4 cells were seeded in a 96-well plate with the density of 5,000/well. Twenty-four hours after transfection the plates were added CCK-8 reagent (DOJINDO Biochemical reagent Company, CK04). Culturing 1 h, then the OD value was measured to indicate cell viability.

### Apoptosis Detection

The apoptosis of EL4 cells were determined by flow cytometry. Twenty-four hours after irradiation of 8 Gy, the EL4 cells were digested and stained using Annexin V-FITC/PI Cell Apoptosis Detection Kit (TransGen Biotech Corp., Ltd, Beijing, China) for 20 min, then flow cytometry was used to analyze apoptosis rate of EL4 cells.

### RNA Extraction and miRNA/mRNA Array

Total RNA was isolated from thymic lymphoma tissues and normal thymus tissues using Trizol (Invitrogen, USA) according to the manufacturer’s instructions (14). Reverse Transcription and qPCR was subsequently performed in triplicate using the mi Script RT Kit and mi Script PCR system (Qiagen). Relative quantities of each miRNA were calculated using the ΔΔCt method after normalization with endogenous reference U6-small nuclear RNA.

Normal and RITL tissues were sent to Capital Bío Company (Beijing, China) for miRNA biochip analysis. The miRNA gene chip used in this experiment is a commercial miRNA oligonucleotide expression profile designed by Agilent based on the miRNA sequence of the database (Sanger miRBase release 16.0: http://microrna.sanger.ac.uk/sequences/, AgilentTechnolo gies) Chip (8×60K). The chip contains probes for human miRNA (1,205 in total) and human virus miRNA (142 in total), which basically covers the currently known human miRNA. This chip is highly sensitive and specific, and can also distinguish mature and precursor miRNAs.

The mRNA chip covers 30,656 human genes and transcripts. It basically covers mRNAs with known functions. The design of each probe is optimized through trial and error, and the average data has more reliable statistical significance, which improves the accuracy of chip detection.

### miRNA/mRNA Microarray Data Analysis

The differential miRNAs were screened using the SAM software from Agilent Inc., which provides a variety of powerful statistical tools that can be used to analyze differential gene expression. This software could normalize the miRNA expression profile data, and then screen the difference genes between groups. Optionally, log2FC and the absolute value of the multiple of the difference between groups were used to represent the multiple of difference. It is required that [log2FC] > 1.0 and P < 0.001, and the two sets of data used for comparison have at least one set of data. Fifty percent of the samples are not Not Detected.

The SAM software was used to normalize the mRNA expression profile data. The difference of mRNA between groups was screened using log2FC and the absolute value of the multiple of difference between groups to represent the multiple of difference, and the filtrated criterion as: (1) p-value ≤0.05 and Fold Change ≥5 in up-regulated mRNAs; (2) p-value ≤0.05 and Fold Change ≤0.2 in down-regulated mRNAs.

### Correlation Analyses of miRNA-mRNA Expression

The MAGIA online software was used to conduct correlation research on the miRNA-mRNA expression. First, the standardized data of the differential miRNA and mRNA is converted into.txt format. In this study, the same sample is used for the detection of miRNA and mRNA chip. Using the MAGIA official website (http://gencomp.bio.unipd.it/magia/start), a Pearson correlation analysis was conducted. calculation for the parameter setting (P < 0.05). Then we can select the miRNA/target stored in the TargetSCan prediction library Gene association database, and imported the txt file. MAGIA software will integrate the miRNA and mRNA differential expression profile chip data to obtain mutually regulated miRNA-mRNA set. The node status and degree distribution of the miRNA-mRNA regulatory network could be analyzed. The distribution of a node refers to the number of adjacent nodes or connected edges that the node catch. The more neighbors associated with a node means that the influence on other factors in the network is more extensive.

### Plasmids

The murine IGF2BP3 over-expression vector was generated by introducing the full length murine IGF2BP3 cDNA into the pcDNA3.1 vector (Invitrogen) in a method like many published papers (13, 15). All PCR products were verified by DNA sequencing.

### Luciferase Assay

Luciferase system was used to detect activity of miR-486 in IGF2BP3 3’ UTR. First, the cells were transfected the suitable plasmid in the 24-well plate. Then, cells were collected to test luciferase assay 48 h after transfection with a reporter plasmid containing IGF2BP3 wt-3’UTR and a plasmid expressing renilla luciferase. Using the luciferase detection kit (e1910, Promega) luciferase detection was performed based on the manufacturer’s protocol (16).

### Enzyme-Linked Immunosorbent Assay

IGF2BP3 secreted from EL4 cells was detected by ELISA. Briefly, the supernate of EL4 cells was collected to measure the contents of IGF2BP3 by ELISA kit (R&D Systems, America). According to the establishment of standard curve, secreted IGF2BP3 level could be quantified.

### Histopathology and Immunohistochemistry

The fixed femur and spleen were thoroughly washed using 0.01 M PBS (pH 7.4) and embedded in paraffin. Paraffin sections cut at a 4 μm thickness for HE. The changes of histopathology were visualized by optical microscope (×200). Immunohistochemistry analyses were performed using paraffin sections. IGF2BP3 expression was investigated. The tissues were incubated in primary antibodies overnight at 4°C and then in horseradish peroxidase (HRP)-conjugated secondary antibodies. Immunoreactive cells were visualized using DAB.

### Functional Annotation

To gain insights into the biological functions of these miRNA target genes, the Gene Ontology (GO) classification was performed. Kyoto Encyclopedia of Genes Genomes (KEGG) was used to analyze the potential pathway of miRNA target genes. The online based software GENECODIS was utilized for those functional annotation (17).

### Tumorigenesis Assay *In Vivo*

48 hours after infection with the ad-virus or negative control, EL4 cells (1 × 10^6^) were subcutaneously injected into the backs of NOD/SCID mice. The tumor formation incidences and the tumor size were measured twice weekly for 30 days.

### Statistical Analysis

Two class unpaired method in SAM was used to analyze expression profile chip. Student’s t-test was utilized to compare the difference between experimental groups and relevant controls. Data were represented as mean ± SD and P < 0.05 was considered significant.

## Result

### miRNAs Expression Profile Analysis and Q-PCR Validation in Radiation-Induced Thymic Lymphoma Tissues of Balb/c Mice

**Figure 1:**
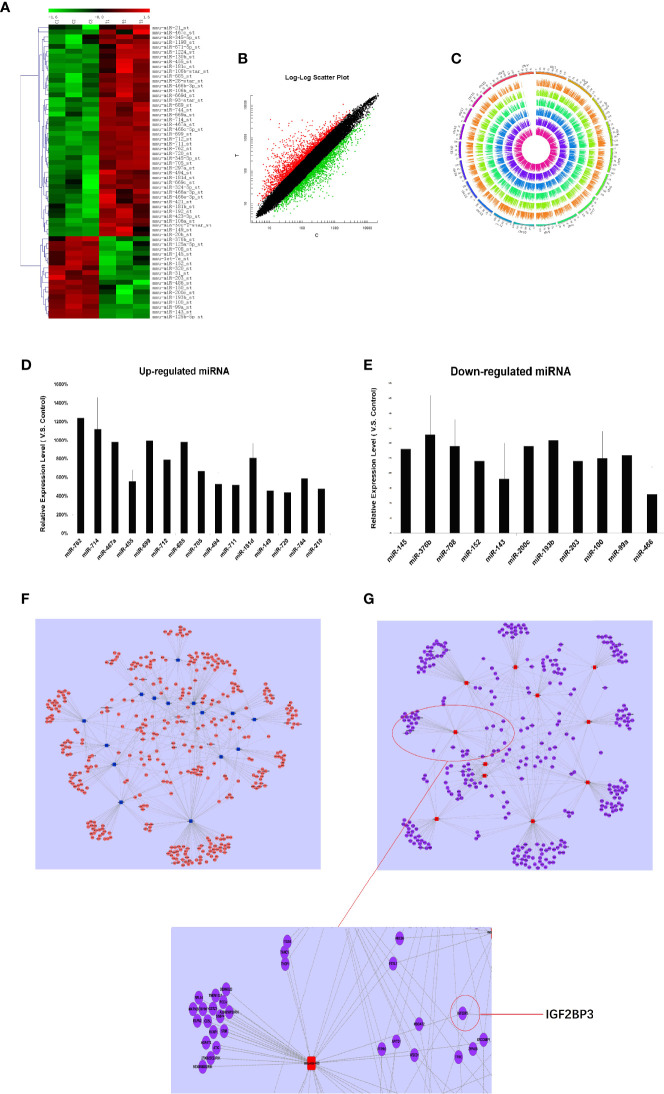
miRNAs expression profile analysis, Q-PCR validation, and establishment of miRNA-mRNA network in RTL tissues of Balb/c mice: **(A)** The miRNAs expression between mouse normal thymus tissues and RTL tissues were primarily screened by using miRNA array (n = 3 mice, each group). Green bar indicates down-regulated miRNA and Red Bar indicates up-regulated miRNA. **(B)** show all differentially expressed mouse genes in RTL tissues (T) *versus* normal tissues **(C)** using mRNA array (n = 3 mice, each group). Red bar: up-regulated genes; Blue bar: down-regulated genes. **(C)** show different locus of different genes on the chromosome, and different color labeling can clearly show the difference information of different groups. The expression of miRNA was verified by Q-PCR. Up-regulated miRNA **(D)** and down-regulated miRNA **(E)** were displayed. Combined the predicted genes and achieved mRNA expression profile, the genuine miRNA targets were finally revealed. Subsequently miRNA target gene pairs with an inverse correlation of expression formed the miRNA-mRNA regulatory network in up-regulated miRNAs **(F)** and in down-regulated miRNAs **(G)**.

Firstly, we detected the miRNA expression in RTL tissue and normal thymus tissue using Affymetrix miRNA chip. Cluster analysis was performed to analyze the result of miRNA chip and demonstrated a significant difference between RTL tissue and normal thymus tissue. Then the SAM software statistical analysis was further used to select objective miRNAs ([log2FC] > 1.0 and P < 0.001). As a result, 63 miRNAs were screened out, of which 44 miRNAs were up-regulated and 17 miRNAs were down-regulated (**Figure 1A**, **Table S1**). Next, the mRNAs expression between RTL tissues and normal thymus tissues were performed by mRNA array. Data were statistical analyzed by SAM software and data were filtrated as criterion: (1) p-value ≤0.05 and Fold Change ≥5 in up-regulated mRNAs; (2) p-value ≤0.05 and Fold Change ≤0.2 in down-regulated mRNAs. As a result, 49 up-regulated and 46 down-regulated mRNAs in RTL tissues were selected (**Figure 1B** and **Table S2**). Further, all these differently expressed mRNAs were located on the corresponding chromosomes. As shown in **Figure 1C**, the three inner circles indicated control groups (normal thymus tissues) and the three outer circles represented RTL tissues. As a result, there were no significant differences between the 20 pairs of chromosomes.

To figure out the biological roles of differently expressed mRNAs, the GO classification enrichment analysis was performed. We found that transcription, DNA templated was significantly enriched in biological processes (**Figure S1A**), cytoplasm was indicated as most enriched cellular component (**Figure S1B**) and protein binding was focused as molecular functions (**Figure S1C**). Besides, KEGG pathway enrichment analysis was performed to indicate significantly difference between RTL tissues and normal thymus tissues. Hypergeometric test with p value <0.05 were used as the criteria for pathway detection. Finally, the most significant pathway was referred in cancer pathway (**Figure S1D**).

Finally, 26 miRNAs were validated by Q-PCR. Compared with the normal thymus tissues, miR-762, miR-714, miR-467a, miR-699, miR-685, miR-181d were most significantly up-regulated in RTL tissues (**Figure 1D**), while miR-143 and miR-486 were most obviously down-regulated (**Figure 1E**), of which miR-486 had the highest fold change.

### Prediction for miRNAs Target Genes and Establishment of miRNA-mRNA Regulatory Network

Based on the screened and validated 26 miRNAs, we conducted their target genes prediction using four bioinformatic algorithms (TargetScan, miRanda, PicTar, miRBase) and a total of 1,070 target genes were obtained. Combined the predicted genes and achieved mRNA expression profile, the genuine miRNA targets were finally revealed. As a result, 761 miRNA target gene pairs with an inverse correlation of expression were formed and miRNA-mRNA regulatory network was accordingly established (**Figures 1F, G**). In this network, miR-486, miR-152, miR-200c, miR-181d, and miR-467 demonstrated the highest connectivity.

### miR-486 Was Potential Tumor Suppressor and Inhibited the Proliferation of Mouse Lymphoma Cells (EL4)

**Figure 2:**
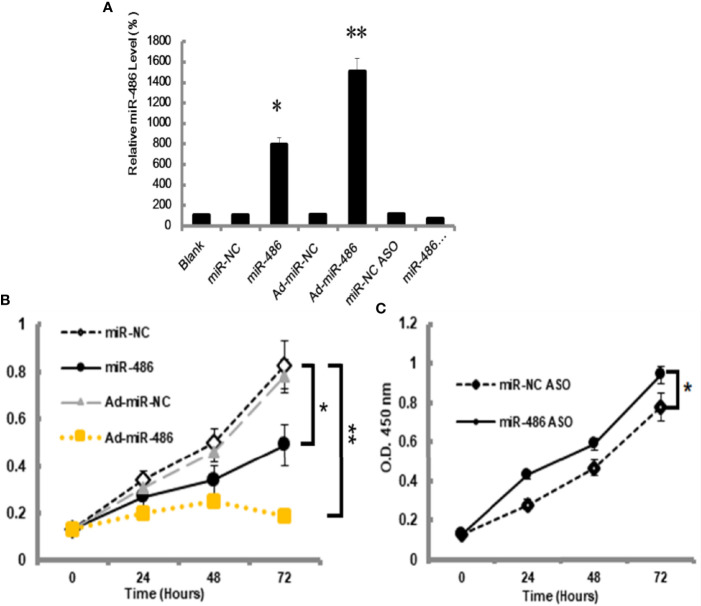
MiR-486 inhibits the proliferation of mouse lymphoma cells (EL4). Synthetic double-stranded DNAs, which code miR-486 mimics, miR-NC mimics (miR-negative control mimics), miR-486 ASO (miR-486 antisense oligo), and miR-NC ASO (miR-negative control antisense oligo), were transfected into EL4 cells. The miR-486 expression was detected **(A)** and cell viability was tested at different time points after transfection **(B, C)**. *P < 0.05, **P < 0.01.

The miRNA-mRNA regulatory network demonstrated that miR-486 had one of the highest connectivity indicating its critical roles in development of RTL. In addition, miRNAs expression profile had revealed that miR-486 was the most down-regulated miRNA. Many studies had confirmed the association between miR-486 and cancer, such as breast cancer (18), hepatocellular carcinoma (19). In this study the role of miR-486 in thymic lymphoma was investigated. The mouse lymphoma cell EL4 was used to study the effect of miR-486 on the proliferation of EL4 cells. By transfection of miR-486 mimics (miR-486) and recombinant adenovirus miR-486 (Ad-miR-486), the expression of miR-486 was up-regulated (**Figure 2A**) and subsequently significantly inhibited the viability of EL4 cells compared with miR-NC group (**Figure 2B**). In contrast miR-486 ASO (miR-negative control antisense oligo) significantly promoted EL4 cells proliferation and rescued their vitality (**Figure 2C**).

### miR-486 Increase Sensitivity of Mouse Lymphoma Cells (EL4) to Irradiation

**Figure 3:**
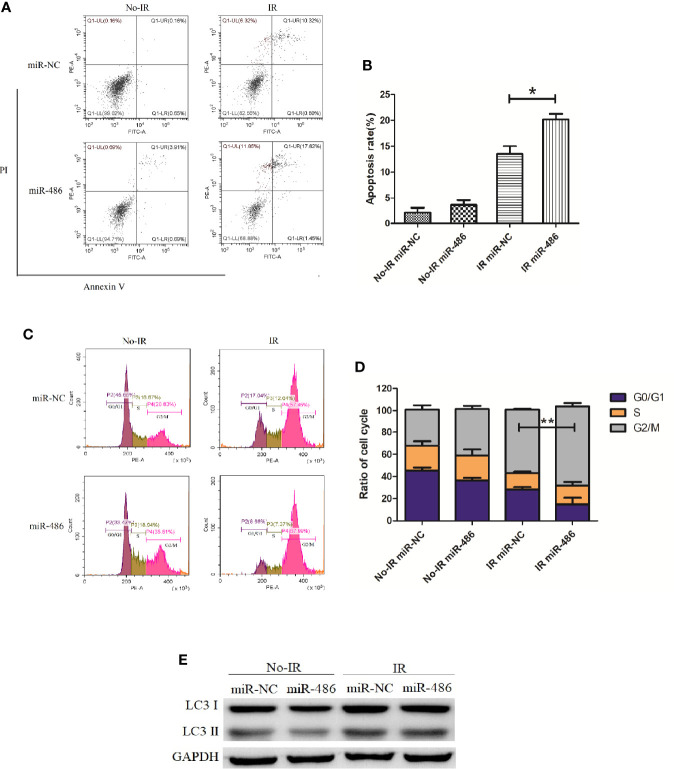
miR-486 increase sensitivity of mouse lymphoma cells (EL4) to irradiation: 24 h after being transfected with miR-486 mimics or miR-NC, EL4 cells were received 8 Gy irradiation exposure. At 24 h, the apoptosis of EL4 cells were detected by flow cytometry **(A, B)**. Twelve hours after irradiation, the cell cycle was analyzed by flow cytometry **(C, D)** and the expression of LC3 was detected to indicate autophagy by western blotting **(E)** *P < 0.05, **P < 0.01.

Next, we investigated the effect of miR-486 on radiation sensitivity of EL4. Here, we mainly studied the effects of miR-486 on apoptosis, cell cycle, and autophagy after irradiation exposure, which are the important radiobiological effects. Twenty-four hours after being transfected with miR-486 mimics or miR-NC, EL4 cells were received 8Gy irradiation exposure. The autophagy, cell cycle, and apoptosis were detected at 12 and 24 h, respectively. The results showed that irradiation can induce apoptosis (**Figures 3A, B**), cause G2/M phase arrest (**Figures 3C, D**), and increase cell autophagy (**Figure 3E**, conversion of LC3 I to LC3 II increases). miR-486 significantly up-regulated apoptosis (**Figures 3A, B**) and increased cycle arrest (**Figures 3C, D**), and increase cell autophagy (**Figure 3E**, conversion of LC3 I to LC3 II increases) after irradiation exposure, but it had no obvious effect on cell autophagy (**Figure 3E**). These results indicated that miR-486 can not only inhibit the proliferation of EL4, but also increase its sensitivity to radiation, suggesting that miR-486 plays an important role in radiation-mediated tumor injury.

### IGF2BP3 Was Identified as miR-486 Target

At present, it is believed that miRNA plays a critical biological function by targeting specific mRNA and subsequently degrading it. Previous experiments had suggested miR-486 as pivotal regulator in RTL development. Further, we try to find the target mRNA of miR-486 to clarify involved mechanism. Prediction for miRNAs target genes had involved 37 genes for miR-486 (**Table 1**), of which IGF2BP3 had the highest fold change (3.87), which reminded us that miR-486 might work by targeting IGF2BP3 genes.

**Table 1:** Target genes corresponding to miR-486 by miRNA-mRNA association analysis.

microRNA	Ratio_microRNA	mRNA_Symbole	Ratio_mRNA	Database source	
mmu-mir-486	0.17	RTKN	3.62	Sanger	
**mmu-mir-486**	**0.17**	**IGF2BP3**	**3.87**	**Sanger**	
mmu-mir-486	0.17	ITGA9	2.12	Sanger	
mmu-mir-486	0.17	TANC1	2.27		TargetScan
mmu-mir-486	0.17	SKP2	2.05	Sanger	
mmu-mir-486	0.17	9530058B02RIK	2.82	Sanger	
mmu-mir-486	0.17	AGPAT2	2.84	Sanger	
mmu-mir-486	0.17	FARSB	2.12	Sanger	
mmu-mir-486	0.17	RPL14	2.58	Sanger	
mmu-mir-486	0.17	MICAL3	2.3	Sanger	
mmu-mir-486	0.17	2700023E23RIK	2.83	Sanger	
mmu-mir-486	0.17	FSTL1	2.29	Sanger	
mmu-mir-486	0.17	PCCA	2.19	Sanger	
mmu-mir-486	0.17	XRCC5	2.04	Sanger	
mmu-mir-486	0.17	STOML2	2.26	Sanger	
mmu-mir-486	0.17	BIVM	3.6	Sanger	
mmu-mir-486	0.17	TREX1	2.72	Sanger	
mmu-mir-486	0.17	ELFN2	2.05		TargetScan
mmu-mir-486	0.17	SRM	3.43	Sanger	
mmu-mir-486	0.17	PSRC1	2.96	Sanger	
mmu-mir-486	0.17	IMPDH2	2.07	Sanger	
mmu-mir-486	0.17	DENND2D	2.4	Sanger	
mmu-mir-486	0.17	THOP1	2.65	Sanger	
mmu-mir-486	0.17	CAD	2.97	Sanger	
mmu-mir-486	0.17	TMEM132A	3.12	Sanger	
mmu-mir-486	0.17	SSBP4	2.06	Sanger	
mmu-mir-486	0.17	ABCB6	2.47	Sanger	
mmu-mir-486	0.17	ATIC	2.69	Sanger	
mmu-mir-486	0.17	SERPINF1	2.37	Sanger	
mmu-mir-486	0.17	A230050P20RIK	2.12	Sanger	
mmu-mir-486	0.17	DYX1C1	2.05	Sanger	
mmu-mir-486	0.17	PYCR2	3.18	Sanger	
mmu-mir-486	0.17	GSTZ1	2.39	Sanger	
mmu-mir-486	0.17	DCLRE1A	2.12	Sanger	
mmu-mir-486	0.17	NIF3L1	2.24	Sanger	
mmu-mir-486	0.17	CTDSPL	2.96		TargetScan
mmu-mir-486	0.17	WSCD1	2.01	Sanger	

**Figure 4:**
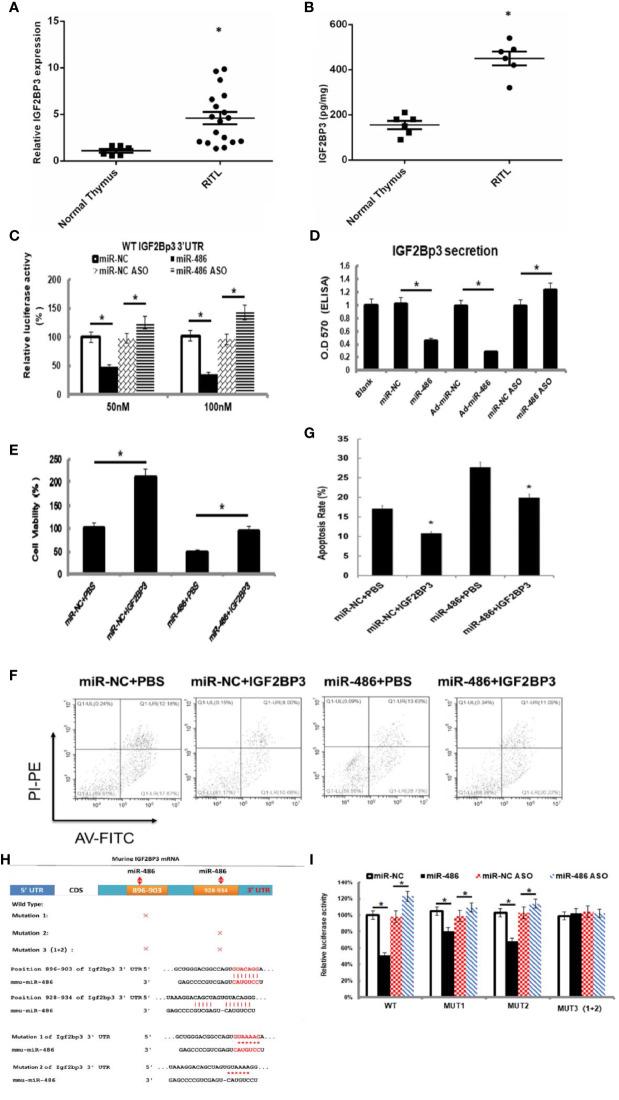
IGF2BP3 was identified as miR-486 target: Prediction for miRNAs target genes had involved 37 genes for miR-486 (Table 3), of which IGF2BP3 had highest fold change (3.87). Thus, the role of IGF2BP3 in RTL was investigated. Using the RTL tissues and normal thymus tissues, their expressions of IGF2BP3 mRNA and protein were detected by QT-PCR and ELISA respectively **(A, B)**. EL4 cells were transfected with synthetic double-stranded DNAs, which code miR-486 mimics, miR-NC mimics (miR-negative control mimics), miR-486 ASO (miR-486 antisense oligo), and miR-NC ASO (miR-negative control antisense oligo). At 48 h after transfection a reporter plasmid containing IGF2BP3 wt-3’UTR and a plasmid expressing renilla luciferase were co-transfected into EL4 cells. Luciferase activities were measured at 48 h after transfection with the plasmids **(C)**; Secreted content of IGF2BP3 from EL4 cells **(D)** and cells viability **(E)** were also detected by ELISA and CCK-8 assay respectively. With the irradiation (8 Gy) the apoptosis of EL4 cells was induced. Then 24 h after irradiation exposure the apoptosis rate of EL4 cells was detected by flow cytometry **(F, G)**. Predicted wild type (WT) and mutant (MUT) miR-486 binding sites at mouse IGF2BP3 mRNA 3’UTR **(H)**. Forty-eight hours after transfection with a reporter plasmid containing IGF2BP3 mut-3’UTR and a plasmid expressing renilla luciferase, the luciferase activities of EL4 were measured **(I)**. *P < 0.05.

To verify above speculation, the mRNA and protein expression of RTL tissues were detected by RT-PCT assay and ELISA respectively. The results demonstrated that mRNA and protein of IGF2BP3 were highly expressed in RTL tissue compared with normal thymus tissue (**Figures 4A, B**). Next, Luciferase (DLR) assay was carried out to test the IGF2BP3 mRNA activity. A mouse 3 ‘UTR of IGF2BP3 gene named p-P16 YUTR was established and consequently miR-486 was confirmed to inhibit the 3 ‘UTR activity of IGF2BP3 mRNA (**Figure 4C**). ELISA verified the reduced IGF2BP3 secretion from EL4 cells transfected with miR-486 mimics (miR-486) and recombinant adenovirus miR-486 (Ad-miR-486), while miR-486 ASO (miR-486 antisense oligo) rescued the secretion of IGF2BP3 (**Figure 4D**). To further confirm IGF2BP3 effect, we measured the viability of EL4 cells and detected apoptosis induced by 8 Gy of irradiation. The results demonstrated that over-expression of miR-486 could significantly inhibit the viability and increase radiation-induced apoptosis of EL4 cells, while added recombinant IGF2BP3 could rescue EL4 cell viability and reduce apoptosis (**Figures 4E–G**). Above results fully demonstrate that IGF2BP3 is the target of miR-486, and miR-486 can inhibit the proliferation of lymphatic cancer cells by inhibiting the expression of IGF2BP3.

## Combined Sequences of miR-486 Targeting IGF2BP3 3 ′UTR Region

Information retrieval demonstrated that miR-486 contained two complementary sequences within IGF2BP3 mRNA 3’UTR. To further clarify the functional sequences, we performed IGF2BP3 mRNA 3’UTR mutation vector experiments with construction of one wild-type and three 3’UTR mutant vectors of IGF2BP3 gene in which MUT1 was 899th C into A, and the 901bpG will be replaced by A; MUT2 was 931bp C into A, and the 933bp G for A; MUT3 was MUT1 and MUT2 combination (**Figure 4H**). The results showed that both MUT1 and MUT2 could weaken the inhibition of miR-48 to IGF2BP3 3 ‘UTR region, and MUT1 was stronger than MUT2 (**Figure 4I**), which indicated that miR-486 plays a role by inhibiting both two complementary sequences (896-903bp and 928-934bp) in the 3 ‘UTR region of IGF2BP3.

## The Adenovirus Over-Expression miR-486 Vector Reduced Tumorigenesis in Vivo

**Figure 5:**
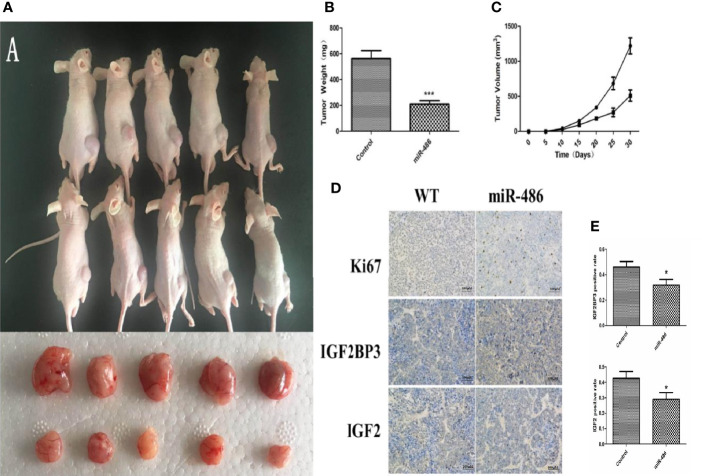
Effect of over-expression of miR-486 on tumorigenesis in nude mice. After inoculation with EL4 cells, the tumor weight and volume were measured **(A–C)**. By immunohistochemistry analysis, the expression of IGF2BP3 and IGF2 were detected **(D, E)**. *P < 0.05, ***P < 0.001.

To evaluate the effects of miR-486 on tumorigenicity, EL4 cells were infected with an adenovirus vector of miR-486 to over-express miR-486. As described above, the validation of increased miR-486 level was done (data not shown). Then, 1 × 10^6^ infected EL4 cells were injected into the back of five NOD/SCID mice 48 h after adenovirus vector infection, and cells infected with the adenoviral vector were similarly injected into the control mice. We examined all mice with lymphoma and found that the EL4/Ad- miR-486 cells formed smaller tumors than these in the EL4/Ad-NC group (**Figures 5A, B, C**). To understand the effects of miR-486 on cell proliferation, we used Ki67 staining to evaluate the proliferation of tumor and found that tumors proliferation was significantly decreased in the miR-486 group compared with the NC group (**Figure 5D**). Then we detected the levels of IGF2BP3 and IGF2 by using immunohistochemistry assay. The results showed that the number of IGF2BP3+ and IGF2+ cells per screen was decreased in miR-486 group compared with the NC group (**Figures 5D, E**). These results suggested that miR-486 exhibited a strong suppressive effect on lymphomagenesis *in vivo*.

## MiR-486 Knockout Mice Have A Strong Tendency of Radiation-Induced Carcinogenesis

**Figure 6:**
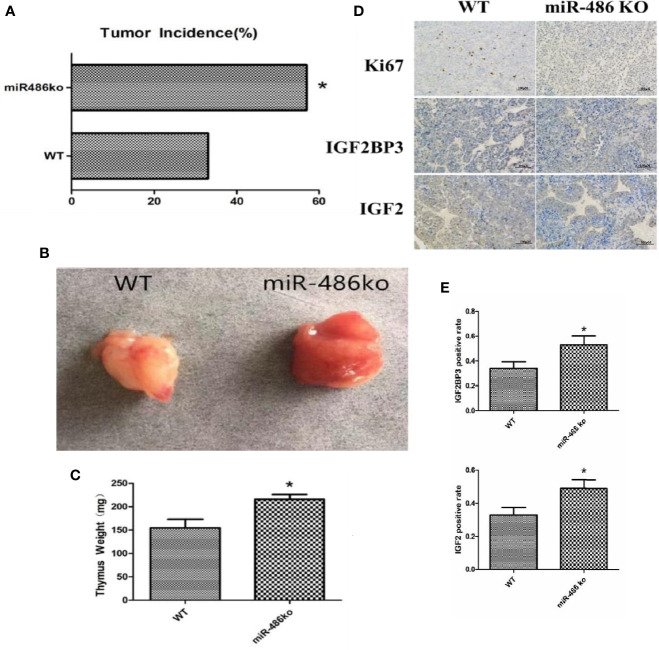
MiR-486 knockout mice has a strong tendency of radiation-induced carcinogenesis. MiR-486 knock out mice were subjected to ^60^Co γ-ray whole body irradiation with fractional dose of 1.75 Gy. The total irradiation dose was 7.0 Gy dividing into four times and dose rate approached to 0.58 Gy/min. The occurrence of radiation-induced carcinogenesis was observed and recorded **(A)**. Subsequently, the tumors were taken to compare volume and weight **(B, C)**. By immunohistochemistry the expression of IGF2BP3, IGF2, and Ki67 were detected **(D, E)**. *P < 0.05.

In order to better study the role of miR-486, we established miR-486 knockout mice. The RTL model and ^60^Co γ-ray whole body irradiation were described in our previous work. Compared with the wild-type mice, the tumor incidence of miR-486 knockout mice were significantly increased, indicating that miR-486 knockout promoted the occurrence of radiation carcinogenesis (**Figure 6A**). The tumor volumes of miR-486 knockout mice were larger than those of wild-type mice, which indicated that miR-486 knockout could promote tumor growth *in vivo* (**Figures 6B, C**). The immunohistochemistry results showed that the positive rate of Ki67 in tumor tissues of miR-486 knockout mice was significantly higher than that of the control group, indicating that miR-486 knockout could promote tumor proliferation (**Figure 6D**). And the positive rates of IGF2BP3 and IGF2 were also higher than that of wild type mice, indicating that by miR-486 knockout, the inhibitory effect on IGF2BP3 was significantly attenuated, and the expression of IGF2BP3 could promote the expression of IGF2 (**Figures 6D, E**).

## Involved Signal Pathways of miR-486 Targeting IGF2BP3 in Regulating Radiation Carcinogenesis

**Figure 7:**
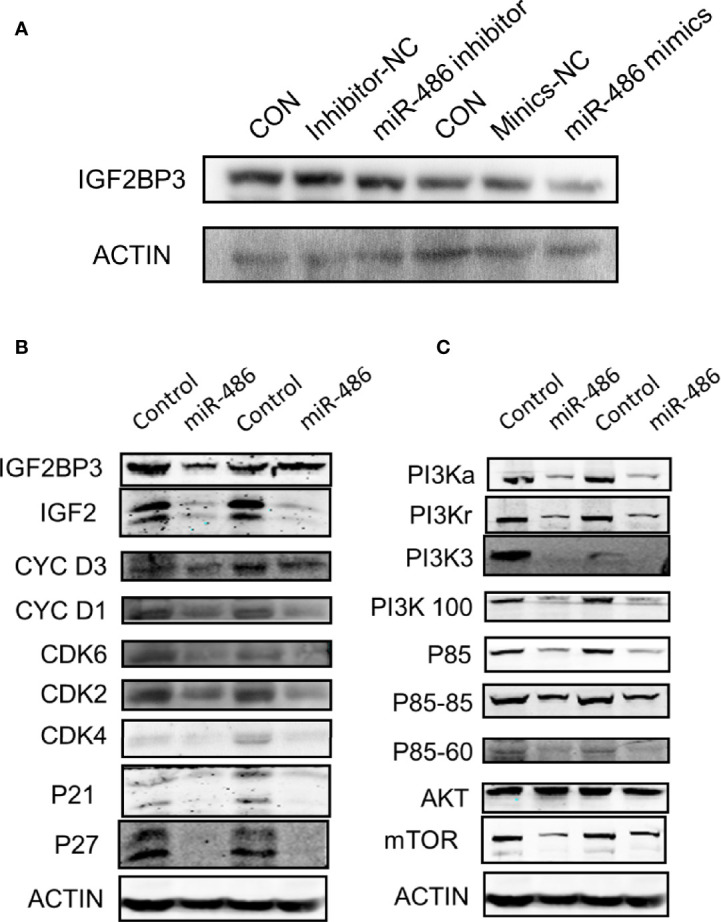
Involved signal pathways of miR-486 targeting IGF2BP3 in regulating radiation carcinogenesis. With miR-486 inhibitor and mimics transfection, the expression of IGF2BP3 was detected by western blotting **(A)**. Besides, proteins of cell cycle pathway, apoptosis pathway and P53 signaling pathway, were also detected **(B, C)**.

Based on the determination of miR-486 targeting IGF2BP3 to regulate radiation carcinogenesis, we further explored its possible downstream signaling molecular pathways. According to the results suggested by our previous GO analysis of mRNA expression profile (**Figures S1A–C**) and KEGG pathway (**Figure S1D**) analysis, the cell cycle pathway (**Figure S2A**), apoptosis signaling pathway (**Figure S2B**), and P53 signaling pathway (**Figure S2C**) were indicated as the most significant signaling pathway to regulate radiation-induced carcinogenesis. To validate above results, we further investigated the expression of IGF2BP3, IGF2, PI3Ka, PI3Kr, PI3K3, PI3K100, P85, AKT, and mTOR (these proteins were important indicators of cell cycle pathway, apoptosis pathway and P53 signaling pathway) in EL4 cells with/without miR-486 mimics by western blotting experiments. As a result, miR-486 mimics down-regulated the expression of IGF2BP3 (**Figure 7A**), concomitantly inhibit the expression of cell cycle related proteins D1, D3, CDK2, CDK4, CDK6, P21, P27 other proteins (**Figures 7B, C**), indicating that miR-486 may inhibit the proliferation of EL4 cells by regulating the cell cycle.

## Discussion

With the wide application of nuclear energy, ionizing radiation exposure becomes increasingly common. The atomic bombings are the most dangerous environmental factor while it does not happen easily. Epidemiological studies of the survivor of the Hiroshima and Nagasaki atomic bombings provide strong evidence that high doses and high dose rates of radiation increase the risk of solid cancers and leukemia. In addition, increased risk has also been demonstrated in cohorts of radiation workers who have been exposed to lower doses of radiation generally and over extended periods of time. Radiation-induced carcinogenesis is one of the late effects of ionizing radiation and is also the nuclear great concern from professionals and people (2). Since 1999 our laboratory began to study the mechanism of radiation-induced tumor and successfully established fractional irradiation induced thymic T lymphoma model in Balb/c mice. Using this mice model, we identified and validated some genes involved in RTL development. However, most of these studies mainly focused on genes and proteins.

Recent studies showed that the expressions of miRNAs are closely related to tumor occurrence, development, metastasis, and prognosis (8, 9), but there are few reports on radiation induced carcinogenesis. Through the analysis, screening, and validation of miRNA expression profiles, our team reported the roles of miR-21 and miR-467 in radiation-induced mouse thymus T lymphoma in the early stage (10, 11). MiRNAs play biological roles by targeting corresponding mRNAs. One miRNA could target multiple genes, meanwhile one mRNA could also be regulated by several miRNAs (12). Thus, the miRNA-mRNA regulation forms a complicated crosstalk network, which perplexes searching critical miRNAs. Benefited by high-throughput screens and bioinformatic analysis, in recent years, we have approaches to effectively detect the target miRNAs or genes from the complex, interlocking, and interacted network (20).

Firstly, miRNA expression profile analysis was performed to screen out 63 differential expressed miRNAs, and 26 miRNAs were validated by Q-PCR assay. Compared with the normal thymus tissues, miR-762, miR-714, miR-467a, miR-699, miR-685, miR-181d were the most significant up-regulation in RTL tissues, whereas miR-143 and miR-486 were obvious down-regulated (21–23), and we had verified the up-regulation of miR-467 in RTL (11). Despite the function of these miRNAs have been proven, miR-486 demonstrated the highest fold change in down-regulation suggesting its critical role in development of RTL.

Next, the mRNAs expressions in RTL tissues were investigated with mRNA array and 49 up-regulated and 46 down-regulated mRNAs in RTL tissues were identified. Besides, to clarify the functions of these differential expressed mRNAs, the GO classification and KEGG pathways analysis were performed. Then the transcription, DNA templated was significantly enriched for biological processes, and cancer pathway was recommended as most significant pathway. This analysis forcefully indicated that the differential expressed mRNAs are related to cancer development.

Based on the screened and validated 26 miRNAs, their target genes were predicted. Combined the predicted genes and achieved mRNA expression profile, the genuine miRNA targets were finally revealed. A total of 761 miRNA target gene pairs with an inverse correlation of expression formed the complicated miRNA-mRNA network. As expected, the network displayed that miR-486 was just located in critical regulatory point. Therefore miR-486 was selected as the research objective. Actually, miR-486 has been reported as a powerful regulator in the process of tumor growth (24), metastasis (25), and recurrence (26). However, the role of miR-486 in radiation-induced carcinogenesis remains unknown.

Next, by using mouse lymphoma cells (EL4), we test the biological function of miR-486 and found that overexpression of miR-486 could significantly inhibit the proliferation of EL4 cells, confirming that miR-486 is a critical cancer suppressor. We investigated the target mRNA of miR-486 by using miRNA-mRNA correlation analysis, and found that 37 associated mRNAs were involved, in which IGF2BP3 was recognized as the most significant gene. Luciferase experiments further demonstrated that the suppressive effect of miR-486 was dependent on IGF2BP3 mRNA 3’UTR activity, verifying that miR-486 targets IGF2BP3 mRNA. Considering that miR-486 contained two complementary sequences combining with IGF2BP3 mRNA 3 ‘UTR, we investigated which one is the functional area of miR-486. With construction of different areas of the mutant IGF2BP3 mRNA 3’UTR region, we demonstrated that both two complementary sequences (896-903bp and 928-934bp) play an inhibitory role in IGF2BP3 expression.

To better understand the function of miR-486, we established adenovirus over-expression miR-486 vector and found that the EL4/Ad- miR-486 cells formed smaller tumors than these in the EL4/Ad-NC group. Moreover, the number of IGF2BP3+ and IGF2+ cells was decreased in miR-486 group compared with the NC group *in vivo*. These results suggested that miR-486 exhibited a strong suppressive effect on lymphomagenesis *in vivo*. In addition, miR-486 knockout mice were established, and RIL model was produced as described. Compared with the wild-type mice, the tumor incidence of miR-486 knockout mice were significantly increased, indicating that miR-486 knockout promoted the occurrence of radiation carcinogenesis. The tumor volumes of miR-486 knockout mice were larger than those of wild-type mice, which indicated that miR-486 knockout could promote tumor growth *in vivo*. The immunohistochemistry results showed that the positive rate of Ki67 in tumor tissues of miR-486 knockout mice was significantly higher than that of the control group, indicating that miR-486 knockout could promote tumor proliferation. And the positive rates of IGF2BP3 and IGF2 were also higher than that of wild type mice, indicating that by miR-486 knockout, the inhibitory effect on IGF2BP3 was significantly attenuated.

**Figure 8:**
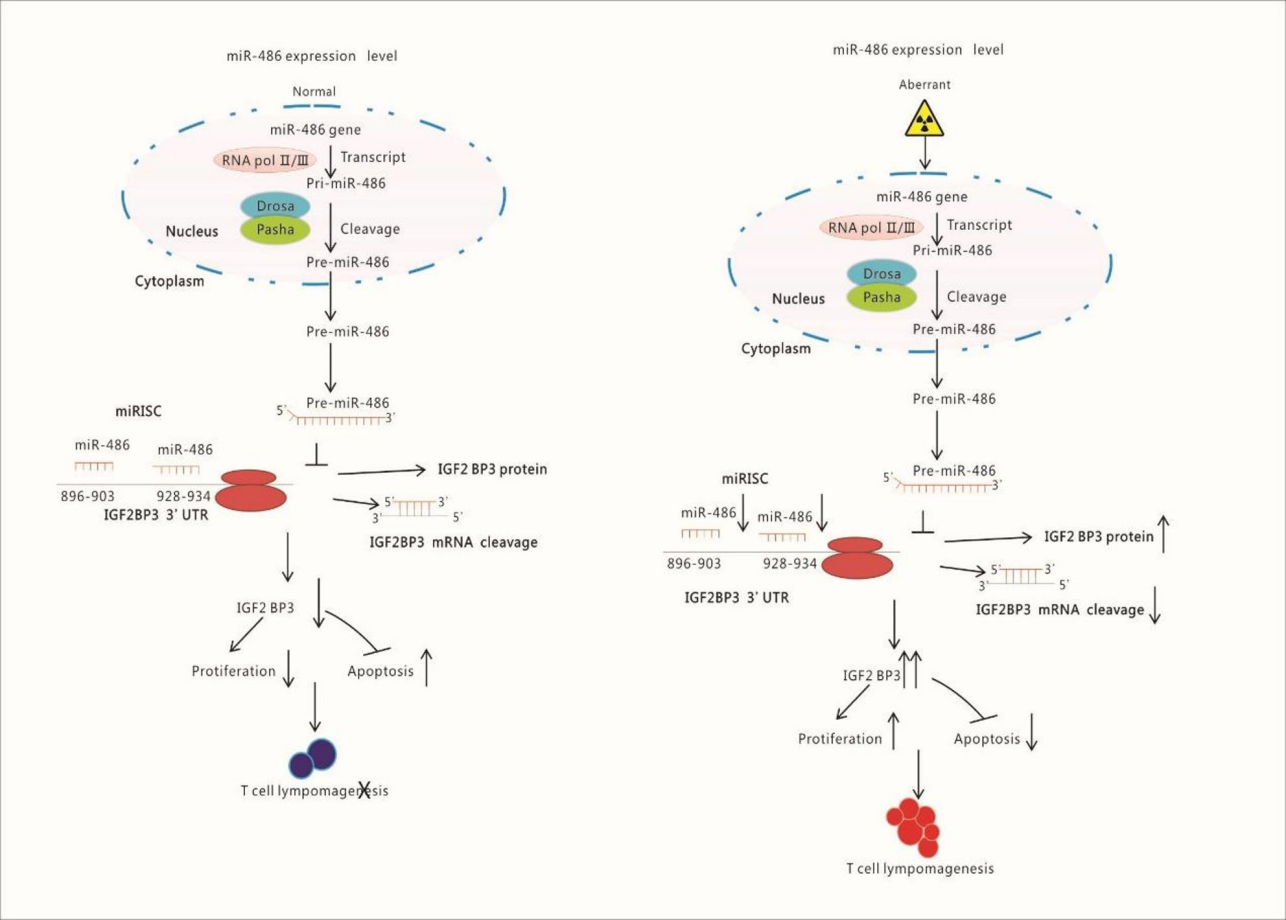
Schematic Illustration of Research Content: Radiation down-regulates the expression of miR-486 which target IGF2BP3. IGF2BP3 is an important molecular to inhibits apoptosis but promotes proliferation of lymphocyte. Finally, the down-regulated miR-486 induces the generation of Thymic Lymphomas by increasing IGF2BP3 expression.

Above experiments and results had demonstrated that microRNA-miRNA network was indeed a novel and effective strategy to find important target miRNA in various of disease including radiation-induced tumorigenesis. Moreover, the role of miR-486 was also confirmed in the development of RTL. Actually, in recent years, many studies have also shown that miR-486 plays a very important role in the occurrence and development of a variety of tumors. For example, Chen H found that the deletion of the miR-486 genome promotes the progression of gastric cancer, and the expression level of miR-486 has a certain reference value for the prognosis of patients with gastric adenocarcinoma (27). In lung cancer, miR-486 was down-regulation and some scholars believe that it promotes tumor invasion and metastasis by targeting ARHGAP5 (25). For other types of cancer, such as breast cancer, cervical cancer, osteosarcoma, and liver cancer, miR-486 was also identified as a critical regulator though their specific mechanism and targets were different (28–31). However, for above studies, the investigation of miR-486 was mainly depended on the expression profile of microRNAs, while rarely using microRNA-miRNA network analysis. Thus, to our knowledge, this is the first study that reporting a complete miRNA expression profile of mouse RTL, moreover, revealing the mechanism of radio-carcinogenesis, which suggested a novel potential therapeutic target for RTL (**Figure 8**).

## Data Availability Statement

The original contributions presented in the study are included in the article/**Supplementary Material**. Further inquiries can be directed to the corresponding authors.

## Ethics Statement

All animal experiments conformed to the National Institute of Health Guide for the Care and Use of Laboratory Animals’ (NIH Publication No. 85-23, National Academy Press, Washington, DC, revised 1996), with the approval of the Laboratory Animal Center of the Second Military Medical University, Shanghai. The approval ID for this study was SMMU-20187321. Written informed consent was obtained from the owners for the participation of their animals in this study.

## Author Contributions

CL, FG, and HL designed the study. HZ, SD, PX, JD, and TL performed the experiments. YY and YC analyzed the data. HZ and HL wrote the paper. JC supported the fund assistance. All authors contributed to the article and approved the submitted version.

## Funding

This study was supported in part by the grants from the National Natural Science Foundation of China (No. 81872559 and No. 11635014) and Shanghai Sailing Program (No. 18YF1429200), Natural Science Foundation of Shanghai (No.18ZR1449700).

## Conflict of Interest

The authors declare that the research was conducted in the absence of any commercial or financial relationships that could be construed as a potential conflict of interest.

## Abbreviations

RTL, radiation-induced thymic lymphoma; EL4, mouse lymphoma cells line; miR-486 ASO, miR-negative control antisense oligo; GO, Gene Ontology; KEGG, Kyoto Encyclopedia of Genes Genomes.
