# Supplementary material for: Analysis of miRNA-mRNA Crosstalk in Radiation-Induced Mouse Thymic Lymphomas to Identify miR-486 as a Critical Regulator by Targeting IGF2BP3 mRNA
Source: Front Oncol. 2021 Feb 22;10:574001. doi: 10.3389/fonc.2020.574001 (PMC7938314; doi:10.3389/fonc.2020.574001)
Supplement: Supplementary file 1 [file DataSheet_1.pdf]

Supplementary Materials for

Analysis of miRNA-mRNA crosstalk in Radiation-Induced Mouse Thymic Lymphomas to identify miR-486 as a critical regulator by targeting IGF2BP3 mRNA

Hainan Zhao[b]#, Suhe Dong[a]#, Jicong Du[a]#, Penglin Xia[a], Ruling Liu[a], Tingting Liu[a], Yajie Yang[c], Ying Cheng[a], Jianming Cai[a], Cong Liu[a]*, Fu Gao[a]*, Hu Liu[a]*

# Hainan Zhao, Suhe Dong, Jicong Du contributed equally to this work

*Corresponding author: Hu Liu, Email: gzsassliuhu@163.com; Fu Gao, Email: gaofusmmu@163.com; Cong Liu, Email: victorliu20102020@163.com

This PDF file includes:

Fig. S1. mRNAs expression in RTL tissues with GO classification and KEGG pathways analysis.

Fig. S2. 3 of the most important signaling pathways involved in the process of RTL.

Tab. S1. All differentially expressed mouse miRNAs in comparisons of T1-T3 VS C1-C3

Tab. S2. Part of differentially expressed mouse genes in comparisons of T1-T3 VS C1-C3

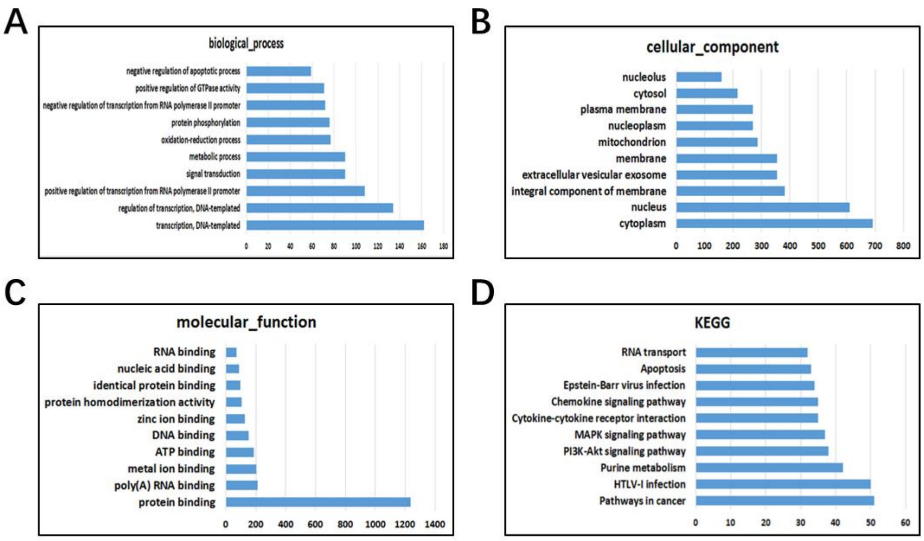

Fig S1. GO Classification and KEGG Pathways analysis of mRNAs: The functional annotation of mRNAs between RTL tissues and normal thymus tissues was conducted by GO Classification and KEGG Pathways analysis. The differentially expressed mRNAs were enriched into biological processes (A), cellular component (B) and molecular functions (C). (D) shows 10 of the most important signaling pathways in which the cancer signaling pathway is most prominent in the RTL organization, which is also very practical.

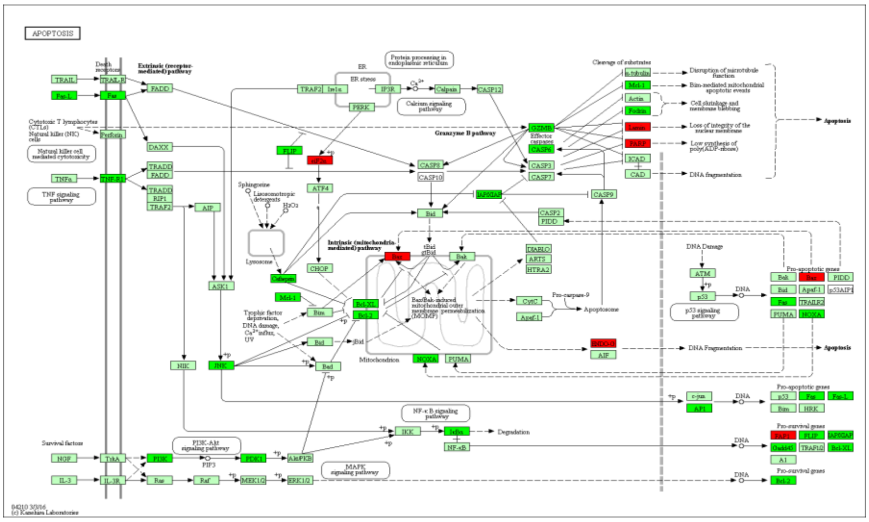

Fig S2-A Apoptosis pathway

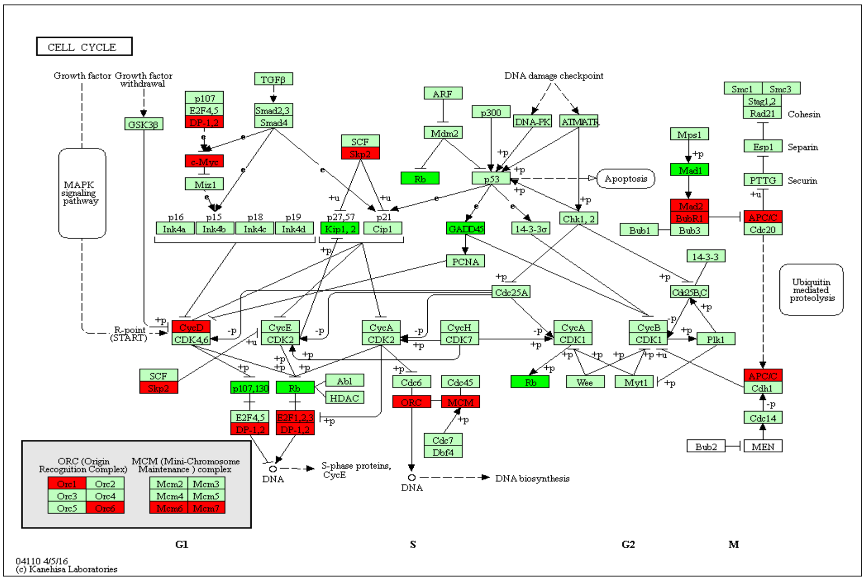

Fig S2-B Cell cycle pathway

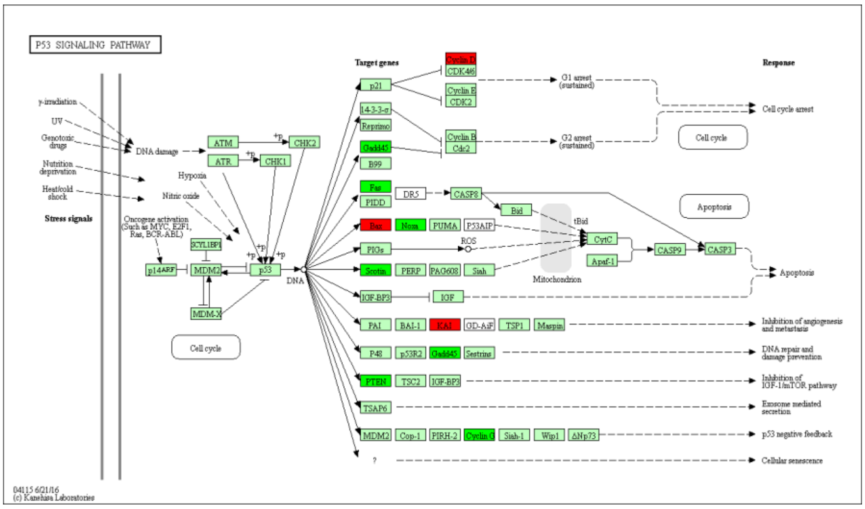

Fig S2-C Cell cycle pathway

Tab S1. All differentially expressed mouse miRNAs in comparisons of T1-T3 *VS* C1-C3

miRNA	Fold Change	C1	C2	C3	T1	T2	T3
mmu-miR-762	16.29995	53.1785	79.0491	56.7184	830.121	1054.67	1195.01
mmu-miR-1224	15.05824	14.6422	28.3890	19.3253	144.937	389.691	404.353
mmu-miR-714	11.84064	25.2979	20.3164	10.0204	205.086	204.814	248.85
mmu-miR-467a	8.993497	39.6074	22.3869	20.2478	236.720	205.876	297.048
mmu-miR-455	8.088613	31.2109	32.9377	25.3580	123.804	398.679	201.501
mmu-miR-699	7.029267	17.4616	15.2747	24.4927	118.171	143.404	140.703
mmu-miR-712	6.428803	18.3208	12.0471	17.2920	89.4958	104.471	112.429
mmu-miR-685	6.162833	88.6207	38.1896	119.005	432.877	601.174	480.871
mmu-miR-705	6.105099	39.0689	38.0218	20.8677	135.672	226.754	235.619
mmu-miR-689	5.986064	11.6245	19.6520	19.2770	103.993	80.2785	118.345
mmu-miR-494	5.65435	34.4538	49.4370	20.3338	308.007	151.878	129.436
mmu-miR-711	4.928502	16.6324	13.1007	15.21	69.6823	67.1815	84.6387
mmu-miR-181d	4.048975	83.2381	103.739	37.3784	361.327	294.601	252.483
mmu-miR-149	3.740021	44.2006	43.2228	37.4694	159.746	231.614	75.7407
mmu-miR-466c-5p	3.560261	25.4234	22.4260	18.7778	81.9984	84.2913	70.9211
mmu-miR-720	3.39201	141.401	148.214	134.330	462.927	446.525	528.578
mmu-miR-744	3.061094	18.2514	24.1779	17.7233	62.7648	46.3654	75.0026
mmu-miR-210	3.054034	85.1111	145.708	117.247	167.083	658.138	237.785
mmu-miR-669c	3.03839	25.5928	40.6563	12.7917	95.2017	68.0033	76.9521
mmu-miR-671-5p	2.939634	18.9173	11.1667	13.6123	26.1858	56.3387	45.9271
mmu-miR-466b	2.840524	14.3840	12.5811	20.5821	43.6671	51.8091	39.5830
mmu-miR-93	2.759742	20.7872	31.2408	23.6820	75.2095	75.2292	58.5013
mmu-miR-130b	2.71546	341.970	325.832	359.930	638.742	1072.91	1079.11
mmu-miR-21	2.711959	45.8795	38.8022	15.8378	58.5999	93.9895	120.015
mmu-miR-345-5p	2.616764	29.9252	19.4661	59.4024	82.6259	124.592	77.4696
mmu-miR-28-star	2.608482	23.2207	14.1079	22.9167	44.647	58.1214	54.3806
mmu-miR-324-5p	2.59365	40.3607	46.3655	19.2582	88.0344	98.6218	88.2304
mmu-miR-466a-3p	2.584312	16.2989	17.1604	10.2015	43.8735	26.3014	42.6582
mmu-miR-181c	2.553082	56.5037	55.5920	68.6313	115.924	208.044	137.442
mmu-miR-181b	2.510714	1697.41	952.985	860.377	3528.40	3001.48	2284.65

mmu-miR-467c	2.371236	17.7430	16.7794	14.8608	39.3922	22.7937	54.9135
mmu-miR-345-3p	2.366719	14.0309	17.4256	11.4777	31.7484	33.6383	36.2266
mmu-miR-466e-3p	2.342553	22.1523	19.5949	15.4438	59.0403	33.9539	40.9791
mmu-miR-192	2.337433	25.5656	18.5268	13.8254	43.5146	56.3697	35.4949
mmu-miR-106b	2.335864	457.508	382.235	496.206	950.837	1208.90	960.854
mmu-miR-1198	2.316914	27.6248	20.5274	40.0138	70.7933	62.7140	70.7661
mmu-miR-106a	2.188859	675.125	563.677	641.697	1415.55	1457.60	1242.99
mmu-miR-106b	2.184321	102.695	98.5000	111.266	167.298	303.598	211.620
mmu-miR-669a	2.171295	16.2217	27.3903	19.9955	48.6795	40.9565	48.4748
mmu-miR-297a	2.126719	25.1735	26.7657	21.7732	46.4573	54.7988	55.5097
mmu-miR-669d	2.105619	14.9034	9.94151	14.6326	23.0809	35.0980	24.9458
mmu-miR-30c	2.038946	14.1193	12.242	12.0391	29.9951	25.7418	22.5595
mmu-miR-421	2.028943	22.8130	21.2772	16.3328	50.6702	30.8309	41.0939
mmu-miR-423-3p	2.022929	112.956	85.5128	74.5041	182.305	216.634	153.267
mmu-miR-20b	2.014202	832.639	804.973	775.421	1735.19	1877.51	1247.63
mmu-miR-20b	2.013521	13.6491	12.3863	20.0198	31.8858	43.0954	17.7520
mmu-miR-320	0.470245	162.356	167.129	129.259	63.3613	81.4806	70.8803
mmu-let-7e	0.456293	371.518	512.564	498.025	152.590	186.752	291.304
mmu-miR-31	0.438599	91.2414	69.5214	89.6808	31.8596	38.3885	39.5963
mmu-miR-150	0.42951	489.47	438.772	436.658	277.347	134.451	174.443
mmu-miR-145	0.423382	690.768	992.685	944.353	316.297	374.508	421.759
mmu-miR-125a-5p	0.397347	211.183	253.218	217.857	74.6584	62.1876	134.247
mmu-miR-376b	0.367487	216.377	383.566	439.559	101.424	154.294	126.284
mmu-miR-708	0.363598	60.0487	69.8726	73.9306	23.0969	18.7564	32.2668
mmu-miR-152	0.352634	91.5288	158.328	124.199	29.7740	40.9162	61.2146
mmu-miR-143	0.309535	636.801	688.172	629.305	204.821	192.416	207.679
mmu-miR-200c	0.290216	359.078	405.968	344.590	147.720	48.5455	125.768
mmu-miR-193b	0.265919	113.374	167.355	153.267	46.4803	27.1019	41.8259
mmu-miR-203	0.263256	86.3613	46.1323	48.4723	15.2790	16.0102	16.3511
mmu-miR-100	0.231036	93.5010	128.056	111.354	31.7794	17.5401	27.5951
mmu-miR-99a	0.224574	207.403	302.896	204.287	56.6218	57.3419	46.5137
**mmu-miR-486**	**0.172003**	**1225.26**	**1454.16**	**1017.27**	**256.023**	**293.051**	**86.7695**
mmu-miR-125b	0.154761	1718.73	1794.66	1692.97	280.492	231.610	293.642

Red mark indicates up-regulated miRNAs in RTL tissues and Green mark indicates down-regulation. "C" represents control group that is normal normal thymus tissues; "T" represents RTL tissues.

Tab S2. Part of differentially expressed mouse genes in comparisons of T1-T3 VS C1-C3

Gene Symbol	Fold Change	T1	T2	T3	C1	C2	C3
Gm266	260.4477	4271.2417	5640.1011	912.71798	12.715723	9.7900993	9.9974218
Fbp1	61.8923	1207.7365	4856.5568	2172.1313	45.482054	44.898985	26.314815
Pdgfrb	51.8299	983.55009	4055.8402	1914.1685	31.320948	40.883162	42.828917
Col1a2	33.9661	88.411114	2005.2151	2913.9344	17.161091	32.222048	23.84027
Trim2	28.4234	407.53067	1198.0053	175.15596	15.346666	16.419607	14.778726
Spsb4	28.0073	800.37678	973.4989	442.76773	23.23589	22.584869	29.923645
Ripk4	27.2514	1369.1929	203.06926	706.35599	19.005196	22.002647	23.207107
EG667874	26.2544	1125.9344	908.65898	417.49604	25.681892	31.239385	29.4191
Ddc	26.0797	1126.7894	917.93561	1430.0368	39.827739	39.792378	52.614759
Trim2	22.1869	636.38209	1720.7716	360.07674	24.835553	43.333677	33.54632
Emx2	21.7221	69.509618	434.04012	242.45878	8.6898175	9.4512199	8.6898175
Ear11	21.6494	55.383822	391.48428	977.02612	11.087313	13.730056	13.714121
Hdgfrp3	21.1601	1281.211	1298.9344	297.38562	25.220847	51.434399	40.268229
Col1a2	20.5173	339.99871	4291.3582	5733.8908	83.98159	112.97263	102.09484
Pbx3	20.4553	676.6436	920.12073	266.60106	24.664333	30.784582	25.541661
Gm131	20.4465	398.62486	926.8408	163.44756	14.672889	25.276232	19.048693
Zfp503	19.7705	1001.7347	864.25303	1511.3059	47.633154	51.880398	68.514333
Cpsf4l	19.4221	869.32006	1132.9344	546.39088	45.807816	40.828778	39.272828
Arg1	18.7917	153.49808	347.98141	362.23184	15.264449	12.73725	14.996629
Wdr25	18.7675	1022.6011	1617.1128	500.97945	45.55188	68.147554	40.373227
Hdgfrp3	17.3088	1801.4835	1881.5406	346.63431	55.433309	68.286603	59.855682
2700008G24Rik	16.8076	174.32627	279.9344	332.2112	12.826339	16.533927	16.100425
1200009I06Rik	16.7403	747.10833	712.62984	272.26834	24.462776	34.020859	37.128239
Heyl	16.4924	883.69896	1176.2105	141.80943	37.03817	25.443133	34.867617
Ccl8	16.085	3122.5173	3681.4125	3081.2439	309.94325	151.60889	181.12498
Mum1l1	16.0618	111.56357	104.83175	182.2754	9.3458287	8.8661181	6.2088296
Mxra7	15.9612	859.21131	990.33206	598.63558	71.772885	48.45997	36.016372
Trim2	15.7122	390.82952	1081.5344	210.35548	26.216227	30.058828	29.08893
Trim2	14.7648	238.29582	592.27823	113.53876	13.654063	17.819311	20.46206
Sertad4	14.7645	518.53119	232.25101	43.401329	13.052917	11.92664	10.431711

Aldh1b1	14.1856	866.18017	3208.7547	471.99523	93.12193	71.945043	68.593929
Rab34	14.0889	353.89983	956.94962	391.92457	34.720094	32.674604	41.835974
Ifi202b	13.6844	3678.8672	4728.4988	483.60013	124.10106	163.03114	162.25419
Dtx1	13.1509	2517.9344	2405.1639	2169.4912	182.54685	181.70659	174.15517
Cxcl13	13.1001	839.87812	676.96211	199.98024	33.719741	42.929748	34.938255
Ptpn13	13.0557	694.38071	1020.0688	1228.6488	70.556257	77.832289	71.213209
Olfml3	12.6032	331.29315	770.59027	1131.5558	52.479539	43.849842	62.706652
Hes1	12.5858	1013.4099	1008.0545	533.63602	66.795563	56.60496	72.321398
Elavl4	12.4072	405.70433	255.67526	15.155587	9.4512199	9.7444948	8.9372734
Sdc1	12.3095	611.15405	796.80583	540.45519	38.31298	63.521767	57.97954
Rab34	12.2142	221.69192	589.77297	285.99134	24.83712	23.456934	35.222449
LOC100041694	11.9083	542.84825	67.153281	241.81007	19.495749	14.949842	17.910085
Sdc1	11.8825	701.76773	1052.4412	651.39158	55.016542	73.265249	71.141462
Col3a1	11.6777	1041.3036	4849.4949	656.66643	77.934395	134.04424	199.33059
Prmt7	11.3792	2317.447	2871.1588	1922.235	213.36462	190.86626	213.1507
Slc45a3	11.3543	179.97385	387.11276	314.68564	28.71702	21.828953	23.893202
H19	11.3335	24.075063	7995.143	61.680376	19.547898	19.616636	21.267967
Tln2	11.0018	463.76773	280.65413	137.07368	30.006227	17.280432	25.838437
Bmp7	10.7702	417.35106	532.08524	385.44253	39.101832	38.766376	45.19763
Treml2	0.0946	28.03108704	25.30750202	55.43439571	320.5967365	390.4343953	371.5278631
Tiparp	0.0946	68.88878952	68.38389226	48.10728046	591.601062	668.7677287	677.0199327
Calcrl	0.0917	15.63890907	36.86957835	129.7698851	355.0863099	433.752926	629.9343953
Hmgn3	0.0903	11.60994675	10.0788286	28.62748177	164.7406033	154.9269937	178.529699
Gpr174	0.088	13.42644495	13.8881514	23.02741987	195.4781102	176.4711499	182.8369489
St3gal6	0.0879	18.03867867	14.81986288	34.80290726	241.1592801	229.168287	247.8699584
Lypd6b	0.0877	24.66290444	19.31466851	48.95239226	318.737146	367.378346	295.6010132
Bach2	0.0871	40.17932728	49.43440559	49.77801655	506.0822045	578.3769359	510.5529818
Tes	0.0867	52.43439739	49.77613572	139.2660727	691.141747	857.9227717	941.4749662
Arap2	0.0861	29.37951067	19.9319749	57.99373182	326.7856653	394.8104734	412.2783797
Eif2s3y	0.0858	473.5933247	9.871223988	24.33330108	638.2195894	513.601062	550.117434
Dnajc6	0.084	15.29866013	9.927639198	11.56575867	124.5560615	149.4931051	159.008556
Cxcr6	0.082	10.01080048	12.99371696	61.56649026	224.601062	247.4343953	261.7677287
Ly6d	0.0812	53.56731234	47.58375353	189.7677287	1090.767729	919.716475	901.0770002
AW112010	0.0804	62.10106202	102.4343953	471.2677287	2123.82677	1820.12371	1489.767729

Dnajc6	0.0783	149.8855984	26.03693894	53.50285713	697.39574	747.7677287	834.7677287
Ccl5	0.0782	61.4946868	78.12798995	208.9106998	1216.722583	1460.281593	1183.162003
Tctex1d1	0.0769	160.2677287	12.32157827	70.26772868	715.7249161	650.9993301	654.940627
Nebl	0.0756	36.74082254	81.53343571	386.7757008	1246.204533	1401.509624	1537.954999
St3gal6	0.073	12.43755625	16.12016831	17.82809917	198.9914744	206.5852962	223.8733586
Ccnd2	0.0726	20.71626758	47.48686113	90.26772868	526.5371368	760.8521537	579.8028393
Cd69	0.0723	36.89573945	49.23013257	84.43439534	568.601062	767.601062	929.6854286
Ccnd2	0.0716	24.49741741	51.43439889	95.09769027	575.0537271	777.9529714	729.2721404
Tiparp	0.0706	27.74032285	21.08894972	29.5194842	328.5988338	379.8822201	393.2067161
Il18r1	0.0668	24.79732127	29.76026207	88.50928385	501.9040287	731.5547929	596.9852836
2610019F03Rik	0.0668	36.03684742	21.20013148	158.9087977	707.4277305	748.0450242	767.9827369
Ccnd2	0.0654	34.83934474	79.35021457	160.9363557	1079.988187	1218.993983	1208.845939
Ccnd2	0.0644	49.11822147	124.6499699	240.5304103	1603.767729	1855.870715	1854.434395
5830443L24Rik	0.0636	88.34591359	28.0645787	264.2729139	1494.30804	1419.255947	1201.091476
Rag2	0.0631	63.7737682	153.0692755	19.08817773	789.7677287	835.9075374	1121.101062
Ccnd2	0.0589	18.15137414	31.57647413	79.76772868	514.1294179	632.6582276	687.101062
Cdc42ep3	0.0588	81.24821236	15.83749286	70.92441222	672.2355847	797.7057712	836.151814
Klrd1	0.0574	12.89620793	15.21227466	58.24413671	356.2071901	475.4787431	356.6735286
Armcx2	0.0513	13.75880825	12.54578745	74.31426863	479.6596755	414.2640287	478.7432834
LOC665506	0.0499	30.95068713	32.44299119	40.18983202	768.601062	688.4442198	613.7677287
Armcx1	0.0486	14.08090185	17.66544267	19.70766874	341.1415191	343.2456355	364.3540873
Ccr9	0.0483	68.846513	119.1821634	991.3820388	4134.339253	3927.828341	4442.684395
Ccr9	0.0476	39.49142756	69.15583794	584.4230933	2701.529683	2154.290623	2540.860917
Ugt8a	0.0459	6.159042801	275.8018179	6.930008352	412.269012	503.4036048	587.4760133
Gpr174	0.0455	10.02759314	17.06485037	43.99014487	372.0695058	458.3198546	469.3037382
Tcrb-V8.2	0.0438	19.04254901	15.22287772	990.2170438	1459.81797	1404.747226	1671.520596
S100a10	0.0396	42.03521032	61.32172159	128.3269606	1445.449919	1736.761028	2124.804773
S100a10	0.0394	59.81282621	77.43466166	127.2298994	1914.692382	2195.440276	2293.601062
1700021K02Rik	0.0341	12.47447791	16.71297155	34.61759624	437.8454094	581.1573895	713.3086516
Tcrb-V13	0.0264	19.95176963	12.36548994	21.70619148	701.7840597	676.9506898	611.1617623
1700021K02Rik	0.0222	5.042702665	6.658070982	32.77609728	310.3369626	502.500867	642.9040277
Tcrb-V13	0.0188	16.71766433	16.46766878	16.84377157	860.6042366	881.601062	925.9931538

Red mark indicates up-regulated miRNAs in RTL tissues and Green mark indicates down-regulation. “C” represents control group that is normal normal thymus tissues; “T” represents RTL tissues.

Tab S3. Statistic analysis of up-regulated miRNAs

Bonferroni's Multiple Comparison Test	Mean Diff.	t	Significant? P < 0.05?	Summary	95% CI of diff
miR-762 vs miR-NC	1105	8.521	Yes	***	693.5 to 1516
miR-714 vs miR-NC	1035	7.986	Yes	***	624.1 to 1447
miR-476a vs miR-NC	1019	7.863	Yes	***	608.1 to 1431
miR-455 vs miR-NC	441.3	3.404	Yes	*	30.12 to 852.5
miR-699 vs miR-NC	1016	7.839	Yes	***	605.1 to 1428
miR-712 vs miR-NC	686.0	5.291	Yes	***	274.8 to 1097
miR-685 vs miR-NC	882.0	6.803	Yes	***	470.8 to 1293
miR-705 vs miR-NC	602.3	4.646	Yes	***	191.1 to 1014
miR-494 vs miR-NC	458.7	3.538	Yes	*	47.46 to 869.9
miR-711 vs miR-NC	426.7	3.291	Yes	*	15.46 to 837.9
miR-181d vs miR-NC	688.7	5.312	Yes	***	277.5 to 1100
miR-149 vs miR-NC	442.0	3.409	Yes	*	30.79 to 853.2
miR-720 vs miR-NC	415.7	3.206	Yes	*	4.458 to 826.9
miR-744 vs miR-NC	465.0	3.587	Yes	*	53.79 to 876.2
miR-210 vs miR-NC	435.3	3.358	Yes	*	24.12 to 846.5

Tab S4. Statistic analysis of down-regulated miRNAs

Bonferroni's Multiple Comparison Test	Mean Diff.	t	Significant? P < 0.05?	Summary	95% CI of diff
miR-145 vs miR-NC	-67.33	8.748	Yes	***	-91.43 to -43.24
miR-376b vs miR-NC	-62.33	8.099	Yes	***	-86.43 to -38.24
miR-708 vs miR-NC	-70.67	9.181	Yes	***	-94.76 to -46.57
miR-152 vs miR-NC	-74.00	9.614	Yes	***	-98.09 to -49.91
miR-143 vs miR-NC	-80.67	10.48	Yes	***	-104.8 to -56.57
miR-200c vs miR-NC	-71.00	9.225	Yes	***	-95.09 to -46.91
miR-139b vs miR-NC	-66.00	8.575	Yes	***	-90.09 to -41.91
miR-203 vs miR-NC	-73.00	9.484	Yes	***	-97.09 to -48.91
miR-100 vs miR-NC	-73.00	9.484	Yes	***	-97.09 to -48.91
miR-99a vs miR-NC	-72.00	9.355	Yes	***	-96.09 to -47.91
miR-486 vs miR-NC	-85.00	11.04	Yes	***	-109.1 to -60.91
